# Endoplasmic Reticulum Stress Induces Myostatin High Molecular Weight Aggregates and Impairs Mature Myostatin Secretion

**DOI:** 10.1007/s12035-018-0997-9

**Published:** 2018-03-15

**Authors:** Rishibha Sachdev, Karin Kappes-Horn, Lydia Paulsen, Yvonne Duernberger, Catharina Pleschka, Philip Denner, Bishwajit Kundu, Jens Reimann, Ina Vorberg

**Affiliations:** 1 German Center for Neurodegenerative Diseases (DZNE), Sigmund-Freud-Str. 27, 53127 Bonn, Germany; 2 Kusuma School of Biological Sciences, IIT Delhi, Hauz Khas, New Delhi, 110016 India; 3 Department of Neurology, University of Bonn Medical Center, 53127 Bonn, Germany; 4 Department of Neurology, Rheinische Friedrich-Wilhelms-Universität Bonn, 53127 Bonn, Germany

**Keywords:** Sporadic inclusion body myositis, ER stress, Myostatin, Amyloid precursor protein, Protein misfolding, Atrophy

## Abstract

Sporadic inclusion body myositis (sIBM) is the most prevalent acquired muscle disorder in the elderly with no defined etiology or effective therapy. Endoplasmic reticulum stress and deposition of myostatin, a secreted negative regulator of muscle growth, have been implicated in disease pathology. The myostatin signaling pathway has emerged as a major target for symptomatic treatment of muscle atrophy. Here, we systematically analyzed the maturation and secretion of myostatin precursor MstnPP and its metabolites in a human muscle cell line. We find that increased MsntPP protein levels induce ER stress. MstnPP metabolites were predominantly retained within the endoplasmic reticulum (ER), also evident in sIBM histology. MstnPP cleavage products formed insoluble high molecular weight aggregates, a process that was aggravated by experimental ER stress. Importantly, ER stress also impaired secretion of mature myostatin. Reduced secretion and aggregation of MstnPP metabolites were not simply caused by overexpression, as both events were also observed in wildtype cells under ER stress. It is tempting to speculate that reduced circulating myostatin growth factor could be one explanation for the poor clinical efficacy of drugs targeting the myostatin pathway in sIBM.

**Electronic supplementary material:**

The online version of this article (10.1007/s12035-018-0997-9) contains supplementary material, which is available to authorized users.

## Introduction

Sporadic inclusion body myositis (sIBM) is the most prevalent acquired muscle wasting disorder above the age of 50 with no effective treatment or cure [1–3]. Patients with sIBM exhibit slowly progressing weakness and atrophy of skeletal muscles [4]. sIBM is regarded as the most enigmatic disease in myology, if not neurology [5]. Pathological characteristics include vacuolated muscle fibers, deposition of proteinaceous material [6, 7], and T lymphocyte infiltration [8]. sIBM shares the immunological signs of an autoimmune attack by cytotoxic CD8-positive T cells in skeletal muscle fibers with polymyositis [9]. The presence of autoantibodies supports autoimmune activity [10]. Immunosuppressants and anti-inflammatory drugs fail to improve the overall course of disease progression, suggesting that inflammation might not be the primary cause of sIBM [11]. Typically, muscle fibers show immunoreactivities for various proteins associated with inclusions in neurodegenerative diseases [12–16]. Protein deposits sometimes stain with Congo Red, indicative of ordered protein aggregates, so-called amyloid [17]. One aggregation-prone protein implicated in sIBM pathology is the amyloid precursor protein (APP). In Alzheimer’s disease, sequential cleavage of APP in the secretory pathway or within the endosomal system leads to the accumulation of Aβ peptides in the brain [18]. APP or metabolites thereof sometimes also form intrafiber deposits or cluster around sIBM vacuoles, suggesting similar pathogenic events [12, 19]. The finding that unrelated proteins aberrantly deposit during disease suggests that pathogenesis is the consequence of protein homeostasis collapse [20]. The pathogenic cascade that leads to proteostasis breakdown is unknown.

Several lines of evidence argue that endoplasmic reticulum (ER) stress plays a prominent role in sIBM pathology [21, 22]. ER stress can be evoked by a variety of stimuli, including energy deprivation, inflammatory stressors, and aberrant expression of unfolded/misfolded proteins [23]. ER stress activates the unfolded protein response (UPR), a signaling pathway that includes induction of ER stress sensors, upregulation of ER resident chaperones, repressed global protein synthesis and enhanced translation of UPR-target genes. Misfolded proteins are exported from the ER and subsequently degraded via the ubiquitin proteasome system (UPS) or autophagy [24]. Calcium dysregulation [25], upregulation of ER-resident chaperones [21, 26–28] and UPR transcription factors [22], suppressed translation [25] and altered clearance of proteins by UPR and the autophagosomal-lysosomal pathway are prominent features of sIBM [29–31].

As the cause of muscle wasting in sIBM remains unknown, symptomatic treatment of muscle atrophy has emerged as an alternative strategy for therapeutic intervention. Among the factors that drive atrophic processes in muscle is myostatin (Mstn), a member of the TGF-β family that is primarily expressed in muscle and negatively regulates its growth [32]. Conflicting results exist regarding the expression of Mstn precursor protein MstnPP in sIBM. In a recent study, serum levels of circulating myostatin growth factor (Mstn GF) were decreased in some sIBM patients [33]. MstnPP mRNA in sIBM muscle was reported to be up- [33] or downregulated [34]. Expression of MstnPP protein was found elevated in sIBM muscle biopsies [34]. Secreted Mstn GF activates the activin receptor IIB (ActRIIB) receptor complex, thereby altering myogenic expression and resulting in atrophy. Therapies targeting Mstn signaling that aim at blocking the engagement of secreted Mstn GF with its receptor complex have progressed into clinical development [35]. However, anti-ActRIIB antibodies failed to show efficacy in a recent sIBM late phase clinical trial (https://www.morphosys.de), suggesting that Mstn signaling is not a prominent driver of muscle atrophy in this disease. The paradox between serum Mstn GF concentrations, muscular MstnPP mRNA and protein levels observed in sIBM [26] and the lack of a significant effect of anti-myostatin treatment on muscle atrophy is unclear. Downregulation of muscular MstnPP mRNA could by one plausible explanation [33]. Another explanation could be that sIBM-associated ER homeostasis imbalance affects the posttranslational processing of secretory protein Mstn GF. Indeed, histological examination of sIBM muscle biopsies has demonstrated aberrant deposition of MstnPP or its metabolites and areas of intense immunoreactivity for both MstnPP metabolites and APP/Aβ [34]. While the aggregation states of MstnPP and its cleavage products have not been assessed biochemically in vivo or in cellular models, amyloid formation of human MstnPP has recently been demonstrated in vitro [36].

We hypothesized that overexpression and ER stress alter the posttranslational processing of MstnPP and also influence Mstn GF secretion. To test this, we employed human muscle cells to study cellular processing of ectopically expressed MstnPP. As a control, we assessed the cellular effects of APP overexpression, also implicated in sIBM disease pathology [28, 37, 38]. We here demonstrate that overexpression of MstnPP but not APP caused misfolding and retention of protein cleavage products in the ER lumen, resulting in dilated ER cisternae and ER stress. Pharmacologically induced ER stress further promoted MstnPP metabolite aggregation into SDS-stable high molecular weight complexes. sIBM histology confirmed aberrant colocalization of MstnPP metabolites and ER resident chaperones, suggesting the involvement of the ER in vacuole and inclusion formation. Importantly, experimentally enhanced ER stress induced MstnPP metabolite aggregation also in wildtype muscle cells and resulted in decreased secretion of Mstn GF. Our data suggest that ER imbalance also impairs mature Mstn secretion in sIBM and question the role of Mstn signaling as a driving force for muscle atrophy in sIBM.

## Materials and Methods

### Patient Samples

Patient samples were collected by the Department of Neurology, University Hospital Bonn. Written informed consent of the patients for the scientific use of residual muscle material was collected as approved by the local ethics committee (069/03 and 070/03), which also approved the specific sample selection and procedures of our study (284/16). Muscle specimens were obtained by standard open biopsy from 20 (4 female, 16 male) patients between 14 and 79 years old (median, 61 years) (Supplementary Table). Muscle biopsies were frozen in melting isopentane and stored in liquid nitrogen. Each underwent standard diagnostic work-up, including: HE, modified Gomori trichrome, ATPase (pH 4.2, 4.6 and 9.4), reduced NADH, periodic acid Schiff, oil-red-O, acid phosphatase, Congo Red, myoadenylate deaminase, phosphofructokinase, succinic dehydrogenase, cytochrome *c* oxidase, and myophosphorylase stainings. Stainings were conducted by standard protocols. Immunohistochemistry, with antibodies against major histocompatibility complex I (MHC-I; 1:1000; W6/32; DAKO), membrane attack complex of complement (MAC, C5b9; 1:100; aE11; DAKO), CD3 (1:50; T3-4B5; DAKO), and CD 68 (1:80; EBM11; DAKO), were included whenever an inflammatory myopathy was clinically suspected or suggested by the standard histology listed above. The respective diagnosis was based on established histological criteria. The control samples were from patients without specific myopathologic changes (e.g., suspected mitochondrial cytopathy cases) or with nonspecific muscular complaints (typically muscle pain or stiffness). Control patients were ultimately declared free of muscle disease. Chronic neurogenic conditions were diagnosed based on fiber type grouping, grouped atrophy, and a bimodal fiber size distribution without major inflammatory or structural pathology as encountered in sIBM. All sIBM samples showed the canonical pathological features [39], i.e., inflammatory myopathy with partial invasion of non-necrotic fibers, rimmed vacuoles, and intracellular congophilic deposits.

### Antibodies and Chemicals

Antibodies were from the following companies; mouse mAb anti-myostatin (MstnPP) (6H12) (Abcam & ThermoFischer Scientific); goat pAb anti-human myostatin (amino acid residues 268–376) (R & D systems); mouse mAb anti-APP 6E10 against Aβ epitope RHDSGYE (BioLegend); mouse mAb anti-APP 22C11 against the aminoterminal residues 66–81 (Merck Millipore); rabbit pAb anti-Giantin ab24586 (Abcam); rabbit pAb anti-LC3B NB100-2220 (Novus biological); rabbit pAb anti-Lamp1 ab24170 (Abcam); rabbit pAb anti-GRP-78 H-129 (Santa Cruz); rabbit pAb anti-GFP A-6455 (ThermoFischer Scientific); rabbit mAb anti-Calreticulin ERP3924 (Merck Millipore); rabbit pAb anti-Calnexin C4731 (Sigma); rabbit pAb anti-Ubiquitin Z0458 (DAKO); mouse mAb anti-Actin (MP Biomedicals); Alexa Fluor-conjugated secondary antibodies (Molecular Probes); HRP-coupled secondary goat antibodies (Dianova). Chemicals were purchased from Sigma or Roth.

### Histological Examination of Muscle Biopsies

Cryostat sections of patient material were studied immunohistochemically according to routine diagnostic techniques. Briefly, 7 μm thick transverse cryosections were transferred onto silaned glass slides, air-dried and fixed in 4% paraformaldehyde for 10 min at RT. Serial sections to those stained for immunohistochemistry were stained with hematoxylin-eosin and modified Gomori trichrome [40] to identify fibers with rimmed vacuoles. Images were captured using × 20–40 objectives and a Nikon H800 microscope (Nikon, Germany) with a SPOT FLEX 64 Mp Shifting Pixel CCD-camera (Visitron Systems GmbH) and SPOT software (version 4.6, Visitron Systems).

### Confocal Microscopy of Muscle Biopsies

Cryosections were fixed in 4% paraformaldehyde in PBS for 10 min at room temperature (RT). Unspecific binding was blocked with 5% BSA and 10% horse serum in phosphate buffered saline (PBS) for 30 min at RT. Muscle tissue was incubated with anti-Mstn 6H12, anti-APP 6E10 or 22C11, or anti-Calreticulin antibodies overnight at 4 °C. Samples were rinsed extensively with PBS and incubated with secondary antibodies for 60 min at RT. After additional washing with PBS, nuclei were counterstained with bis-benzimide (1:10,000 in PBS 0.5 g/ml; Sigma-Aldrich) for 2 min at RT. Specimen were mounted in a Mowiol 4–88 (Calbiochem, Merck Chemicals) and glycerol mix in pH 8.5 Tris buffer with 0.1% DABCO (1,4-Diazabicyclo (2,2,2) octane; Sigma-Aldrich). Confocal laser scanning microscopy was carried out using 40× oil lenses and an LSM 700 laser-scanning microscope (Zeiss). Cross-reactivity of secondary antibodies was excluded by control stainings without primary antibodies (not shown). Single optical planes are shown.

### Cell Lines

The human rhabdomyosarcoma cell line CCL 136 (American Type Culture Collection, Rockville, MD, USA) was used for all experiments. Cells were maintained in DMEM with GlutaMAX (Gibco) supplemented with 10% fetal calf serum (FCS) (Biochrom) and antibiotics. The human MstnPP cDNA (Origene) was used for PCR and the open reading frame was cloned into the lentiviral vector pRRLsin.PPT.CMV.Wpre [41]. For the APP construct, a plasmid encoding the 695 amino acid residue human APP bearing the Swedish mutation (K70M/N671L) (APP-SWE) [42] served as a template to generate a PCR product that was cloned into the lentiviral vector. As a control, the open reading frame for the enhanced green fluorescent protein (EGFP) was cloned into the same vector. Viral particles were produced and cells transduced according to published protocols [41, 43]. Human neuroblastoma cell line SH-SY5Y stably expressing the Swedish mutant of human APP (APP^695^ SWE) [42] was maintained in DMEM, high glucose, GlutaMAX supplement with 10% FCS and antibiotics.

### Western Blot Analysis

Western blot analysis was performed with cell lysates prepared in buffer containing 150 mM NaCl, 50 mM Tris-Cl (pH 7.5), 1% NP-40, 2 mM EDTA and Protease Inhibitor cocktail (Roche). Cell lysates were adjusted to comparable protein concentrations. Proteins were separated on NuPage 4–12% Bis-Tris gels by SDS-PAGE under reducing conditions and blotted onto PVDF membranes by wet blotting.

### Deglycosylation by Peptide N-Glycosidase F (PNGase F)

Deglycosylation by PNGase F was performed according to the manufacturer’s instructions (New England BioLabs). Briefly, 75 μg of total protein in lysates of CCL 136 WT, MstnPP and APP cells was denatured at 100 °C for 10 min and cell debris was pelleted for 10 s. Supernatants were incubated with 1000 units PNGase F at 37 °C for 1 h. Proteins were separated by SDS-PAGE under reducing conditions.

### Sedimentation Assay, SDD-AGE, and Glutaraldehyde Crosslinking

Sedimentation assays were performed with cell lysates prepared in buffer containing 150 mM NaCl, 50 mM Tris-Cl (pH 7.5), 2 mM EDTA, 2% Triton X-100 and complete protease inhibitor cocktail (Roche). Lysates were cleared of cell debris (1000×*g*, 10 min, 4 °C) and subjected to high-speed centrifugation (150,000×*g*) for 1 h at 4 °C. The pellet was resuspended in TNE buffer (50 mM Tris-Cl (pH 8.5), 150 mM NaCl and 2 mM EDTA) and sonicated at 100% amplitude for 2 min (Sonicator Sonoplus, Bandelin). Equal volumes of supernatant and pellet were loaded onto 4–12% Bis-Tris SDS-PAGE gels and proteins were transferred onto PVDF membranes. SDD-AGE was performed according to previously published protocols [44]. CCL 136 MstnPP cells were grown in 6 well plates, while for wildtype CCL 136, cells were seeded onto 10 cm dishes. Briefly, cell lysates were mixed with sample buffer containing TAE, 5% glycerol and 2% SDS (final concentration) for 5 min at RT and separated ON on 1.5% agarose gel containing 0.1% SDS. Proteins were blotted onto nitrocellulose membrane by capillary transfer. For glutaraldehyde crosslinking, cell lysates were incubated with 0.005% glutaraldehyde for 15 min at 37 °C, followed by addition of 50 mM Tris-HCl (pH 8.0) to terminate the reaction. Proteins were separated on 4–12% SDS-PAGE gels and blotted onto PVDF membranes. Antigen-antibody complexes were visualized using ECL Prime (Amersham) for Actin, BIP and overexpressed MstnPP. SuperSignal West Femto Chemiluminescent substrate (Thermo Scientific) was used to detect endogenous MstnPP.

### Immunofluorescence Staining and Confocal Microscopy Analysis of CCL 136 Cells

Cells were grown on coverslips and fixed with 4% PFA for 10 min at RT, and permeabilized using 0.1% Triton X-100 for 5 min. Cells were blocked with 2% goat serum for 1 h followed by incubation with primary and secondary antibodies for 1 h. Nuclei were counterstained with Hoechst for 5 min. Coverslips were mounted in Aqua-Poly/Mount. Confocal laser scanning microscopy was performed using an LSM 700. For automated image analysis, cells were plated on 96 well plates (Greiner Bio-One) and fixed with 4% PFA 48 h post-plating. Cells were stained with anti-APP antibody (6E10) or anti-Mstn-N antibody (6H12). Cytoplasm and nuclei were stained for 10 min with CellMask (1:5000) and Hoechst (1:10000), respectively. Single plane confocal microcopy images were taken using the CellVoyager automated microscope (Yokogawa) with a × 20 objective. Image analysis was performed using the Yokogawa software. As controls, cells were only exposed to secondary antibody. No unspecific antibody binding was detected. APP- or MstnPP-overexpressing cells were determined using a maximum intensity threshold (area > 5 μm) above a local background. GFP cells were detected by counting the number of green nuclei versus total nuclei.

### Chemical Induction of ER Stress

Cells were grown until approximately ~70–80% confluent. Unless otherwise noted, cells were cultured with DMEM containing either 400 nM Thapsigargin (Tg) or 3 μg/ml Tunicamycin (Tm) for 12 h.

### Cell Viability Assay

Metabolic activity of cells was assessed by the XTT assay (Cell Proliferation Kit II, Sigma) according to the manufacturer’s recommendations. Briefly, cells were seeded onto 96-well plates (Greiner) and incubated with the compounds for 12 h. H_2_O_2_ (1 mM) was used as a positive control. Absorbance was measured at 492 and 690 nm 4 h post addition of the XTT reagent.

### ELISA for Cell-Derived Beta Amyloid

Aβ-42 levels in cell lysates and conditioned medium were quantified using Amyloid beta 42 ELISA Kit, Human (ThermoFisher Scientific). Briefly, cells were seeded on 6-well plates and cultured to ~70–80% confluency. The medium was replaced with fresh medium containing DMSO, Tg or Tm. After 12 h, conditioned medium and cells were collected. Cell lysates were prepared as above. The ELISA results were normalized to micrograms of protein in the cell lysates.

### Statistical Analysis

Statistical analysis was performed using unpaired *t* test or one-way ANOVA with Dunnett’s multiple comparison test and GraphPad Prism 6 software, as indicated. All values are expressed as mean +/- SD.

## Results

### Rimmed Vacuoles and Proteinaceous Deposits in sIBM Muscle Biopsies Are Immunoreactive with Anti-MstnPP and Anti-APP Antibodies

**Fig. 1:**
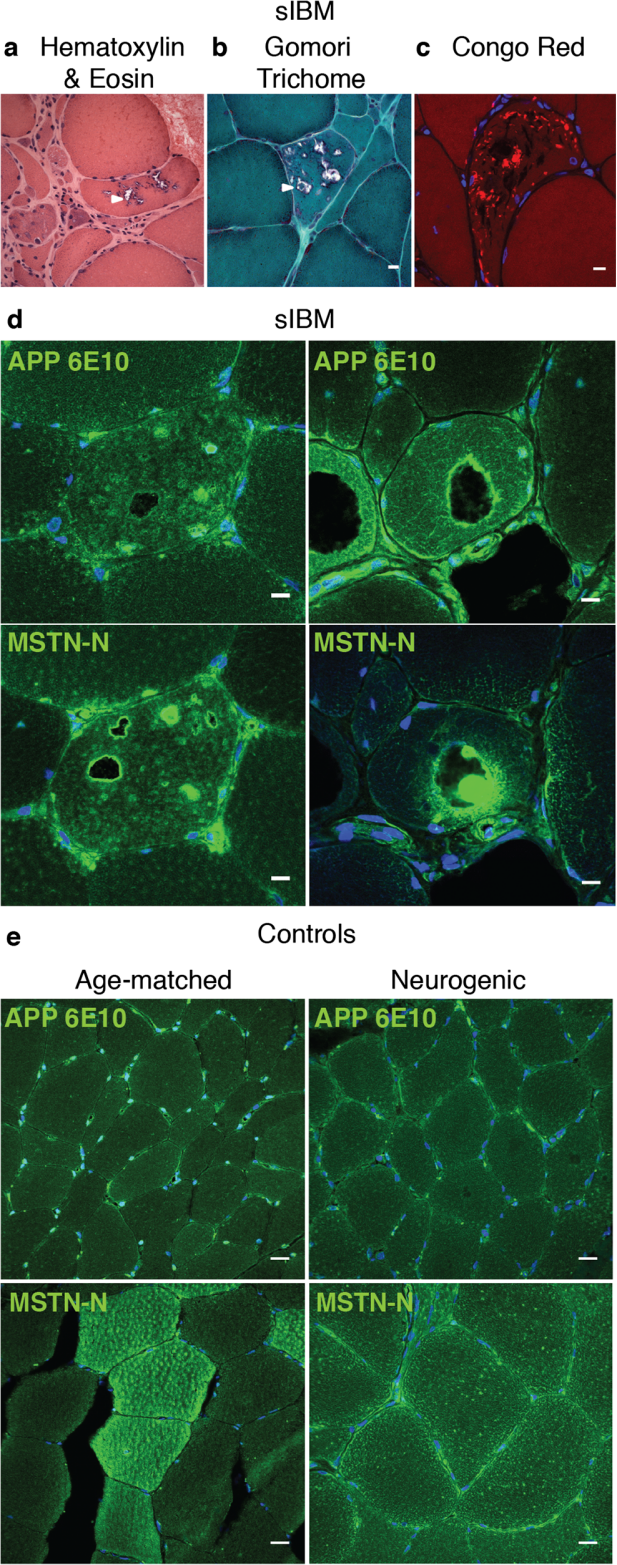
Presence of anti-MstnPP and anti-APP immunoreactivity in sIBM myofibers. **a** Hematoxylin and eosin staining of representative sIBM muscle biopsy, showing variation in muscle fiber size as well degenerative changes, in particular typical rimmed vacuoles (arrowhead) and basophilic sarcoplasmic inclusions. **b** Rimmed vacuoles (arrowhead) stained with modified Gomori trichrome stain. **c** Congo Red fluorescence of myofibers demonstrates sarcoplasmic amyloid deposits, in particular adjacent to vacuoles. One optical plane is shown in all images. **d**, **e** Representative transverse serial sections of skeletal muscle from (**d**) sIBM patients, (**e**) age-matched controls and neurogenic patients. Sections were stained with anti-APP 6E10 directed against the amyloidogenic Aβ region and anti-MstnPP 6H12 directed against the propeptide region of MstnPP (anti-Mstn-N). Nuclei were stained with Hoechst. Scale bars 10 μm

A previous study reported the accumulation of Mstn antibody-positive material in sIBM muscle [34]. To confirm this finding, muscle biopsies from sIBM and controls (Supplementary Table) were assessed histo- and immunohistochemically. Serial sections stained with hematoxylin-eosin confirmed typical rimmed vacuoles in sIBM samples that were frequent in areas of non-atrophic muscle fibers (Fig. 1a). Rimmed vacuoles were present in all sIBM cases but absent in neurogenic controls and controls free of muscle disease (data not shown). Gomori trichrome staining revealed red-rimmed vacuoles characteristic of sIBM (Fig. 1b). The presence of Congo Red positive inclusions confirmed proteinaceous deposits in sIBM skeletal muscle (Fig. 1c). Staining with anti-APP antibody 6E10 binding to the amyloidogenic Aβ region revealed immunoreactivity with inclusions and around vacuole boundaries in sIBM biopsies (Fig. 1d, upper panel).

In skeletal muscle, biologically active Mstn GF is derived from inactive MstnPP. Upon translocation to the ER, disulfide-linked homodimeric pro-myostatin (pro-Mstn) is formed. Further cleavage of pro-Mstn generates a carboxyterminal homodimer (dimer 25 kD, monomer 12.5 kD), complexed non-covalently with its inhibitory aminoterminal propeptide (36 kD) [45, 46]. A second cleavage event in the propeptide disassociates the latent complex [45]. In skeletal muscle, formation of the latent complex and liberation of active Mstn GF occurs predominately extracellularly [47]. We used antibody 6H12 against aminoterminal MstnPP (residues 24–266) (Fig. 1d, lower panel) that detects pro-Mstn, the most abundant MstnPP cleavage product in skeletal muscle [47], and the inhibitory propeptide. For simplicity, we refer to all MstnPP metabolites detected by antibody 6H12 as Mstn-N. Mstn-N often localized around vacuoles in sIBM patient samples (Fig. 1d, lower panel). In some instances, Mstn-N was also found inside of vacuoles or in protein deposits within non-atrophic fibers. In non-myopathic and neurogenic controls, both APP and Mstn-N staining was evident throughout the sarcoplasm (Fig. 1e).

### Co-localization of APP and MstnPP Metabolites with Calreticulin Around sIBM Vacuoles Suggests Involvement of the ER in Vacuole Formation

**Fig. 2:**
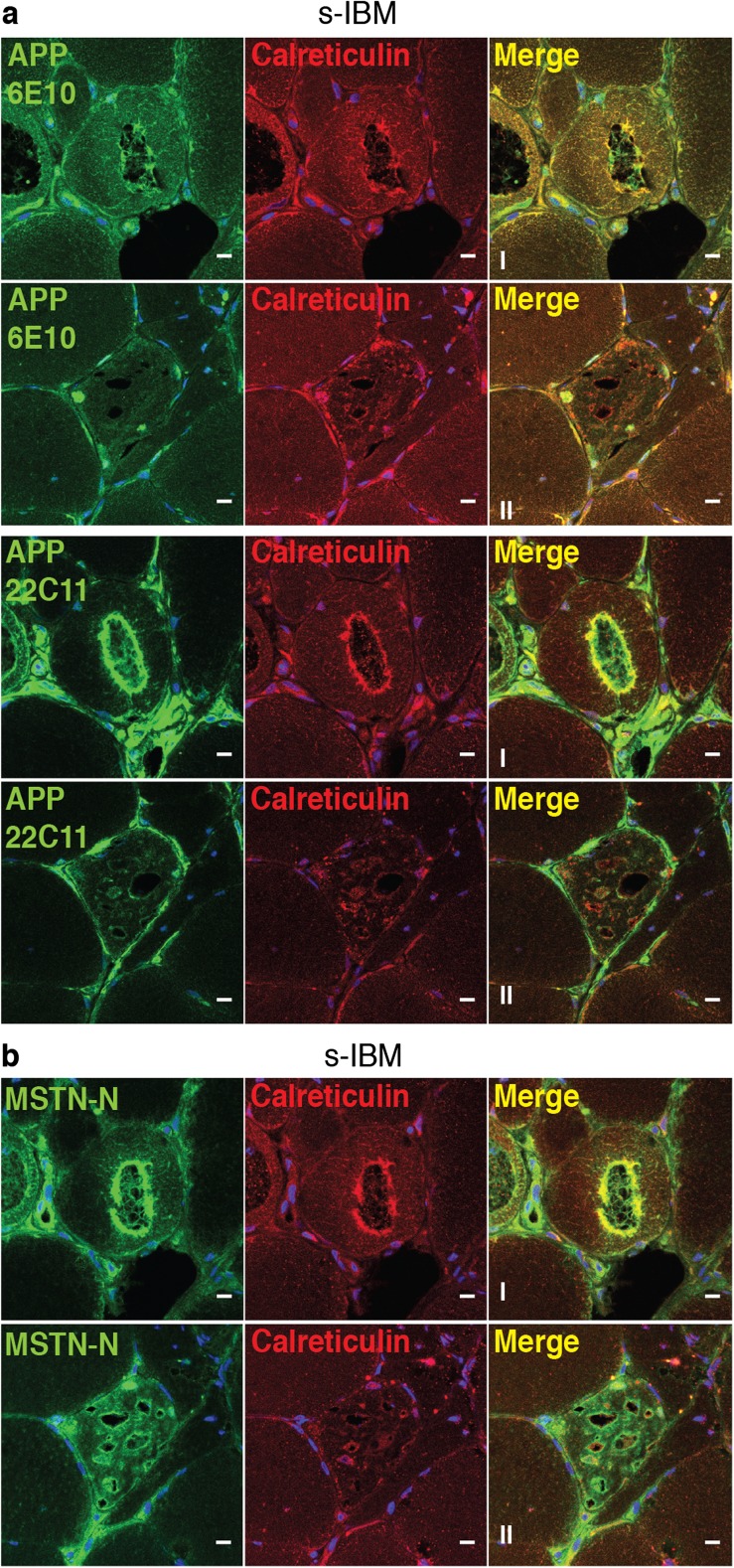
APP and MstnPP metabolites co-localize with ER marker Calreticulin around rimmed vacuoles in sIBM biopsies. Confocal microscopy analysis of representative sIBM patient muscle biopsies co-stained for either (**a**) APP (antibodies 6E10 and 22C11, green) or (**b**) MstnPP (6H12, green) and Calreticulin (red). I and II represent sets of consecutive sections. Antibodies directed against the aminoterminus (22C11) or the Aβ peptide region (6E10) both stain inclusions and boundaries of degenerative vacuoles. Antibody 6H12 directed against Mstn-N reacts with propeptide and pro-Mstn. Prominent staining is found around rimmed vacuoles and co-localizes with Calreticulin. Scale bar 10 μm

Recent electron microscopy examinations of sIBM muscle biopsies revealed arrays of patterned ER associated with rimmed vacuoles [48]. It is thus possible that secretory proteins MstnPP and APP or metabolites thereof were retained within the ER. To analyze the co-localization of MstnPP and APP antibody-immunoreactive protein with ER markers in sIBM tissue specimen, we performed fluorescent double-labeling immunohistochemistry on skeletal muscle biopsies of sIBM patients (Fig. 2). Staining of consecutive sections with APP-specific antibodies revealed that both aminoterminal APP epitopes (antibody 22C11) and the amyloidogenic Aβ region (antibody 6E10) co-localized with Calreticulin at rimmed vacuoles. This argues that full-length APP or aminoterminal APP fragments also deposit in sIBM fibers (Fig. 2a). Notably, antibody 6E10 also detects full-length APP and carboxyterminal cleavage products such as CTF-β, so the presence of Aβ peptides within vacuole walls cannot be revealed using this antibody. Vacuole walls and interior structures of vacuoles consistently stained positive with antibody against aminoterminal MstnPP (Fig. 2b). ER marker Calreticulin also stained intensely around the vacuole walls and interior as small vesicles or puncta (Fig. 2). The finding that both MstnPP and APP metabolites co-localize with Calreticulin supports the hypothesis that the ER is involved in vacuole formation in sIBM.

### Characterization of Human Myoblast Cell Lines Stably Expressing MstnPP or APP

**Fig. 3:**
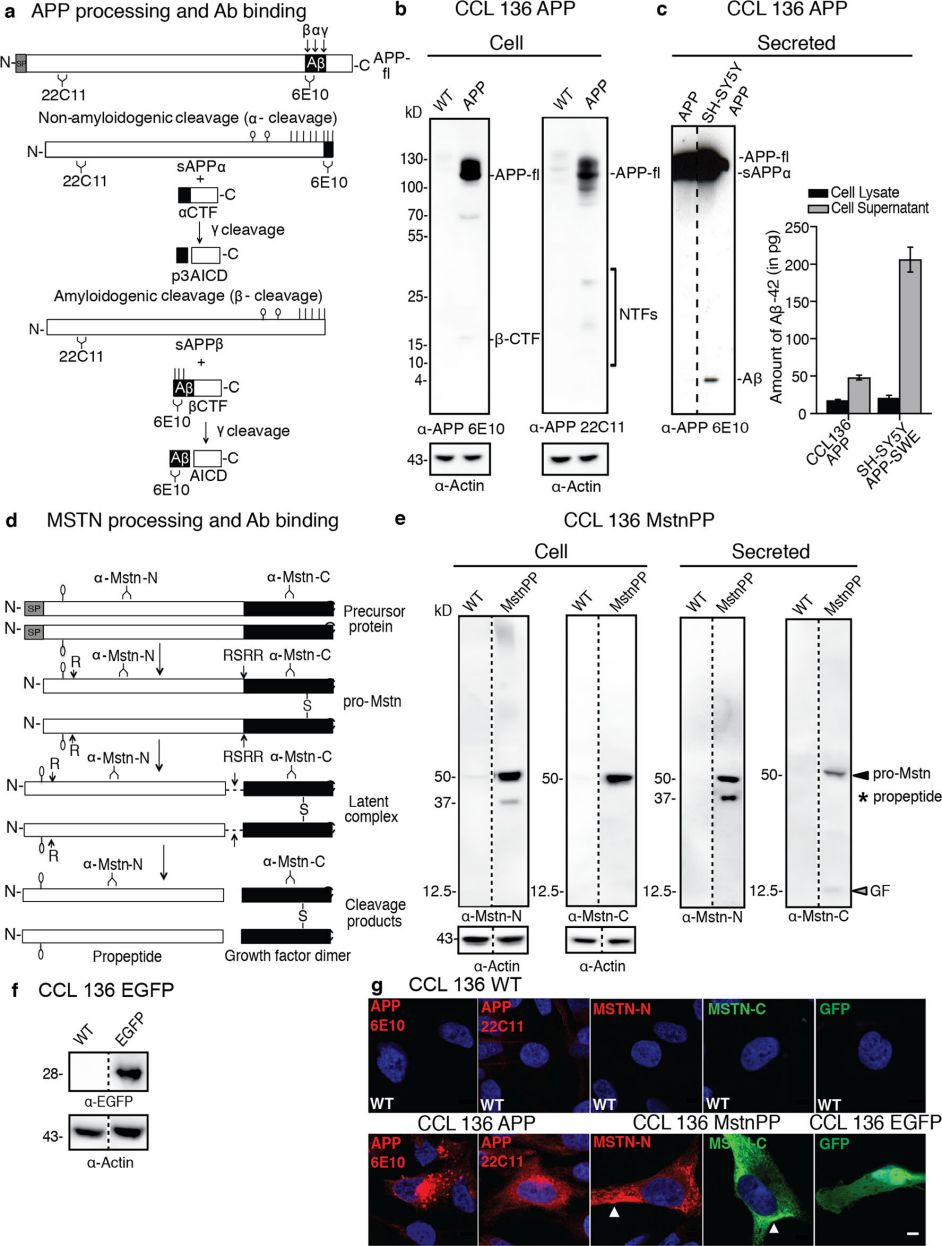
Ectopic expression of MstnPP, APP and EGFP in CCL 136. **a** APP processing. In the nonamyloidogenic pathway, transmembrane APP is sequentially cleaved by α-secretase and γ-secretase. In the amyloidogenic pathway, BACE1 (β-secretase) and γ-secretase generate amyloidogenic Aβ peptides. Signal peptide (SP), antibody binding sites and cleavage sites are shown. Predicted glycosylation sites are marked with lines (O-linked glycosylation) and lines with circles (N-linked glycosylation). **b** APP processing in wildtype (WT) and transduced CCL 136 cells. APP fragments were detected with anti-APP antibody 6E10 (APP residues 597–613) and antibody 22C11 (residues 66–81). APP-fl: APP full length; β-CTF: beta C-terminal fragment and NTF: amino terminal fragment. **c** Western blot analysis of APP fragments secreted into the supernatant of CCL 136 APP cells (antibody: 6E10). Mouse neuroblastoma SH-SY5Y APP-SWE overexpressing APP [42] served as a control. Right graph: Aβ-42 levels detected by ELISA in lysate and conditioned medium (*n* = 3). **d** MstnPP processing in muscle cells. Following SP cleavage in the ER, pro-Mstn undergoes N-linked glycosylation and dimerization via a disulfide bond. The aminoterminal propeptide is cleaved by furin proteases (cleavage site: RSRR) but remains non-covalently bound to the myostatin dimer (latent complex). Subsequent cleavage of the propeptide by metalloproteinases (R) releases mature Mstn GF [46, 53]. Inactive pro-Mstn is the predominant form in muscle [47]. Binding of antibodies is indicated. The exact epitope for antibody 6H12 is unknown. **e** MstnPP processing by CCL 136 cells. Lysate and medium were probed for N- (Mstn-N) and C-terminal (Mstn-C) MstnPP fragments. Pro-Mstn, propeptide and GF are marked by arrowheads and stars. **f** Detection of enhanced GFP (EGFP) overexpressed in CCL 136 cells. **g** Confocal microscopy analysis of transgene expression. Nuclei were stained with Hoechst. EGFP expressing cells serve as expression controls. Arrowheads mark areas of intense Mstn staining. Scale bar 5 μm

Overexpression of APP and MstnPP mRNA and protein has been implicated in sIBM disease progression [34, 49]. Increased expression of secretory proteins could lead to ER stress and impair proper protein processing and trafficking, a hypothesis that we tested in a human muscle cell line. As endogenous expression of both APP or MstnPP is low in cultured muscle cells [13, 50], we transduced the human rhabdomyosarcoma cell line CCL 136 with lentivirus coding for human APP, MstnPP or enhanced green fluorescent protein (EGFP). We expressed APP with the Swedish mutations (K670N/M671L), to generate increased levels of Aβ [51, 52]. Western blot analysis with an antibody against the amyloidogenic Aβ region (α-APP 6E10) or aminoterminal (α-APP 22C11) APP antibodies (Fig. 3a) demonstrated that CCL 136 APP cells predominately expressed full-length APP (APP-fl) and/or sAPP-α of approximately 110–130 kD (Fig. 3b). Faint smaller bands immunoreactive with 6E10 likely represent APP β-CTF (16/17 kD) [18]. Low-abundant aminoterminal fragments (NTFs) (20–26 kD) resulting from aminoterminal cleavage were detected by 22C11 [54]. Aβ peptides were undetectable in western blots of cell lysates or conditioned medium (Fig. 3b, c). Analysis of conditioned medium or cell lysates by ELISA revealed that CCL 136 APP cells produced very low levels of Aβ-42 (Fig. 3c). N-linked glycosylation of APP was verified by Peptide N-glycosidase F (PNGase F) treatment (Supplementary Fig. 1).

Expression of MstnPP was assessed using antibodies against the amino- (6H12) or carboxyterminus (AF788) of MstnPP (Fig. 3d). Antibody AF788 reacts with pro-Mstn and mature Mstn GF, hereafter termed α-Mstn-C. In line with previous reports on mouse myoblasts [50], endogenous MstnPP expression was low and most often undetectable by western blot (Fig. 3e). In lysate of cells ectopically expressing MstnPP, a predominant 50 kD band was detected under reducing conditions, in agreement with the predominance of pro-Mstn in skeletal muscle (Fig. 3e) [47]. A fainter band of approximately 38 kD, detected by 6H12 (α-Mstn-N), corresponds to the liberated propeptide [45]. This band was often not detected in cell extracts by western blot (see subsequent figures). The intracellular reduced monomeric 12.5 kD GF was barely detected. Pro-Mstn, propetide, and GF were also detected in conditioned medium of CCL 136 cells ectopically expressing MstnPP (Fig. 3e). The slight shift in size upon PNGase F treatment demonstrated N-linked glycosylation of pro-Mstn and Mstn propeptide (Supplementary Fig. 1). Western blot analysis verified successful expression of EGFP as a control (Fig. 3f). Transduced cells in bulk populations stained intensely with antibodies directed against N- or C-terminal APP (Fig. 3g). 6E10 and 22C11-reactive protein predominately localized in foci that clustered adjacent to the nucleus, consistent with the APP metabolite presence in the secretory/endosomal pathway forming the perinuclear vesicle cloud [55–57]. Antibodies directed against Mstn-N or -C stained throughout the cytoplasm. Occasionally, areas of more intense staining were apparent (see arrows). EGFP was expressed in both nucleus and cytoplasm.

### Retention of MstnPP Metabolites in ER Cisternae

**Fig. 4:**
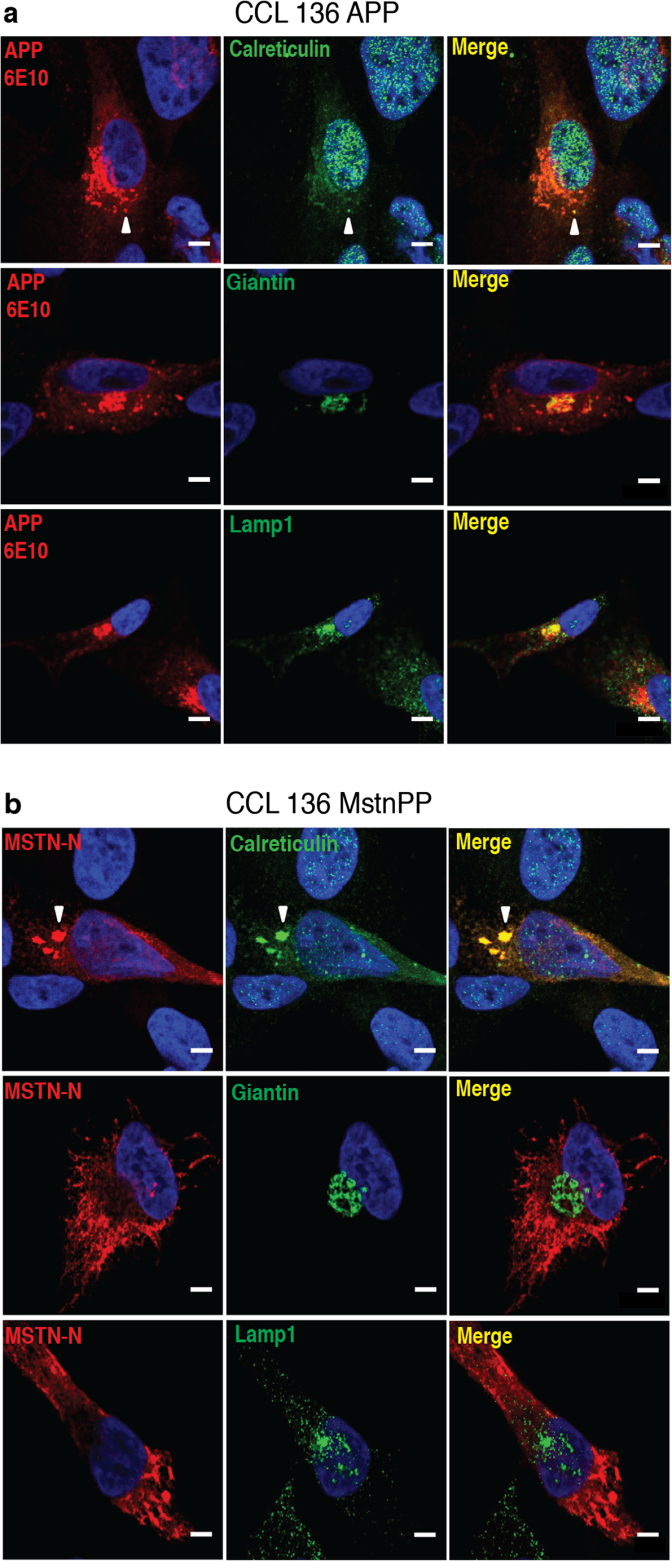
Subcellular localization of APP and Mstn-N in stable CCL 136 cell lines. Cells were stained with antibodies against **a** APP (6E10) or **b** Mstn-N (6H12) (red) and markers for ER (Calreticulin), Golgi (Giantin) and lysosomes (Lamp1) (green). Note the preferential co-staining of Mstn-N with ER over Golgi and lysosomal markers. Arrowheads mark APP or Mstn-N puncta that co-stain with Calreticulin. Nuclei were stained with Hoechst. Scale bars 5 μm

Signal peptides in MstnPP and APP direct the polypeptides to the secretory pathway (Fig. 3a, d). We co-stained cells with MstnPP- or APP-specific antibodies and antibodies directed against marker proteins of ER (Calreticulin, Calnexin, BIP), Golgi (Giantin), lysosomes (Lamp1) and autophagosomes (LC3). 6E10 staining co-localized with ER markers (Fig. 4a, Supplementary Fig. 2a), Giantin-positive vesicles (Fig. 4a), autophagosomes (LC3, Supplementary Fig. 2a) and Lamp1-positive lysosomes (Fig. 4a), consistent with APP’s transit through the secretory pathway and clearance by the degradative system [56, 58, 59]. Anti-Mstn-N antibody intensely co-stained with antibodies against ER markers (Fig. 4b and Supplementary Fig. 2b). Occasionally, the ER architecture appeared distorted, suggesting that overexpression of MstnPP promoted ER swelling. Little to no co-localization was found with Golgi (Giantin, Fig. 4b), autophagosomes (LC3, Supplementary Fig. 2b) or lysosomes (Lamp1, Fig. 4b), arguing that MstnPP metabolites were predominantly retained in ER cisternae.

### MstnPP Metabolites Form SDS-Resistant Assemblies and Cause ER Stress

**Fig. 5:**
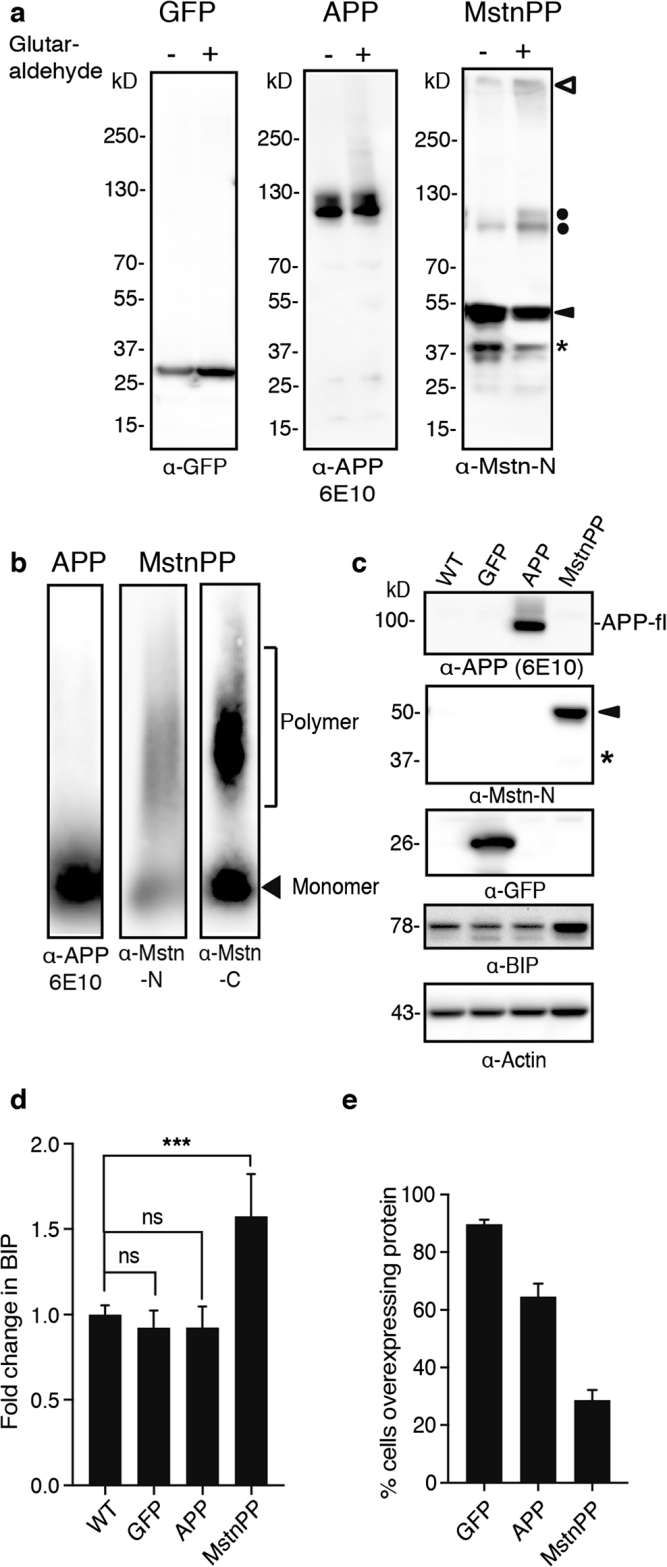
Increased MstnPP metabolites cause ER stress and form SDS-insoluble aggregates. **a** Glutaraldehyde crosslinking of proteins. Lysates were incubated with 0.005% glutaraldehyde and proteins were separated by SDS-PAGE. Pro-Mstn dimers with intact disulfide bonds (dots), high molecular weight species in the stacking gel (white arrowhead), pro-Mstn (black arrowhead) and propeptide (star) are indicated. **b** SDD-AGE analysis reveals presence of aggregated MstnPP metabolites but not APP. Cell lysates were mixed with 2% SDS containing sample buffer and proteins were separated by agarose gel electrophoresis. Arrowhead indicates monomeric, parenthesis indicates polymeric proteins. Please note that molecular weight cannot be determined by SDD-AGE [44]. **c** Western blot analysis of BIP expression in WT and transgenic cell lines. Blots were probed with anti-APP (6E10), anti-Mstn-N (6H12), anti-GFP and anti-BIP antibody. Actin served as a loading control. WT: untransduced CCL 136 cells. **d** Fold change in BIP expression in CCL 136 cells overexpressing MstnPP, APP or EGFP compared to WT controls. Bars represent mean values ± SD (*n* = 4). Statistical analysis was performed using one-way ANOVA with Dunnett’s multiple comparison test. Significant changes are indicated by asterisks ***p < 0.001, ns = not significant. **e** Percentage of cells in transduced bulk populations overexpressing the transgenes as determined by automated confocal microscopy. Fixed cells were stained for APP (antibody 6E10) or MstnPP (6H12). Per cell line, eight wells were plated. Wildtype cells served as negative controls. At least 5000 cells per cell population were counted

Accumulation of protein in the secretory pathway causes ER stress and upregulation of the UPR, both characteristics of sIBM [21, 60]. We assessed the aggregation state of glutaraldehyde crosslinked proteins by western blot analysis. EGFP and APP were mostly monomeric (Fig. 5a). When Mstn dimers were stabilized by glutaraldehyde, anti-Mstn-N antibody 6H12 detected two additional bands at approximately 110–120 kD corresponding to differentially glycosylated pro-Mstn dimers (Fig. 5a, dots). A diffuse smear at the top of the western blot suggested that at least a fraction of 6H12-reactive proteins was present in high molecular weight complexes (Fig. 5a, open arrow). We employed semi-denaturing detergent agarose gel electrophoresis (SDD-AGE) to probe for SDS-resistant polymers [44]. This method dissolves most non-covalently linked protein assemblies, while highly ordered assemblies with cross-beta structure remain intact. APP was almost exclusively present in its monomeric state. Interestingly, when overexpressed, Mstn species immunreactive with both anti-N- and C-terminal MstnPP antibodies were sequestered into SDS-resistant high-molecular-weight assemblies (Fig. 5b). Comparison of BIP levels demonstrated that overexpression of MstnPP significantly increased its expression, indicative of ER stress mediated UPR (Fig. 5c, d). By contrast, BIP levels remained unchanged upon overexpression of APP or EGFP. Increased expression of BIP did not correlate with numbers of cells transduced with the transgenes. Automated confocal microscopy analysis revealed that MstnPP was expressed in only 28.63 ± 3.55% of cells, while GFP and APP were overexpressed in 89.72 ± 0.02 and 64.61 ± 4.58% of cells in the respective bulk populations (Fig. 5e). We conclude that MstnPP overexpression induces ER stress in CCL 136 cells and that Mstn metabolites aggregate into SDS-resistant polymers upon overexpression.

### ER Stress Increases Intracellular Anti-Mstn Immunoreactivity and Induces MstnPP Metabolite Foci Formation

**Fig. 6:**
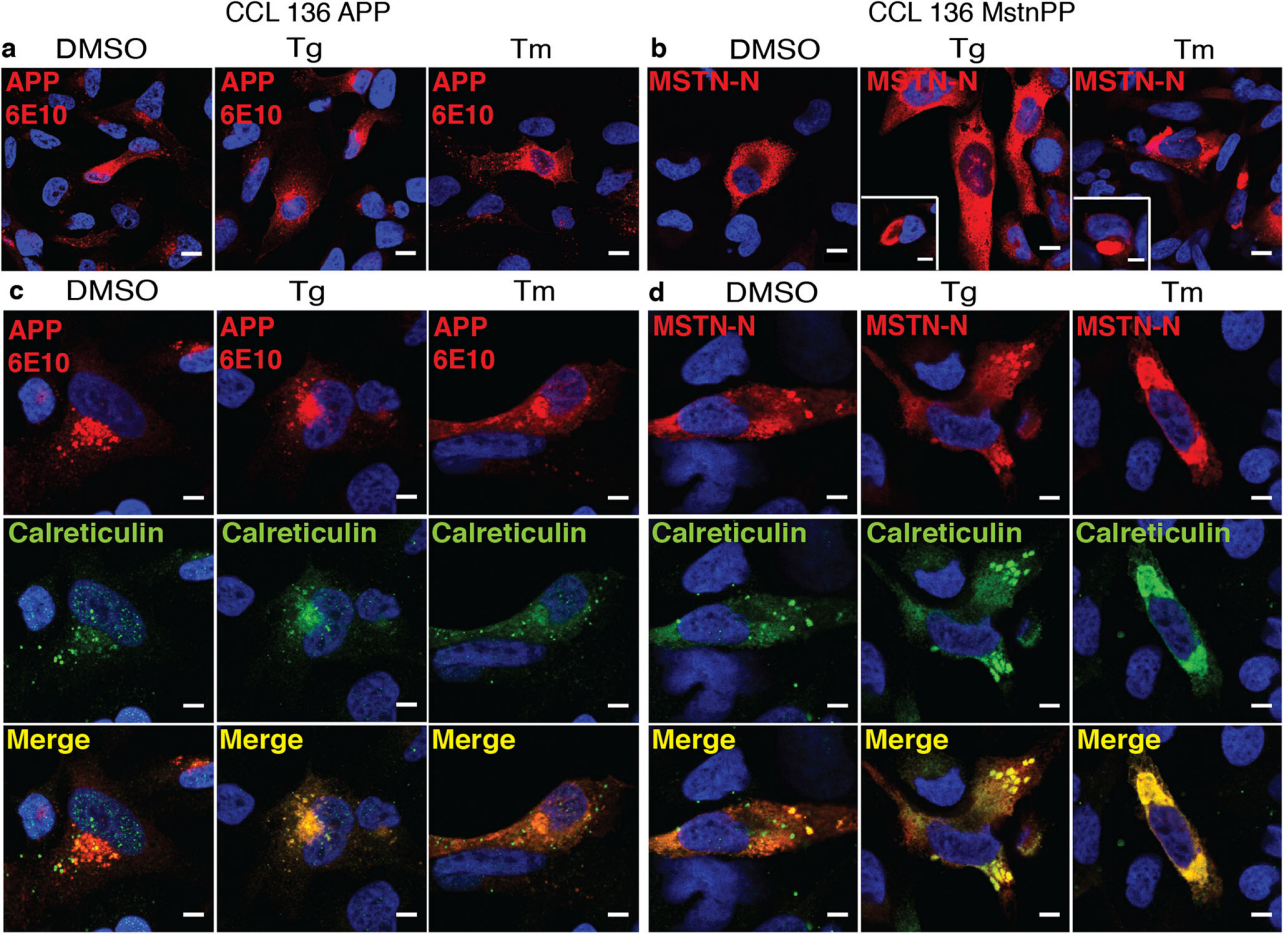
ER stress drives Mstn-N into intracellular assemblies. Confocal microscopy analysis of cells expressing APP and MstnPP upon chemical induction of ER stress. CCL 136 APP and CCL 136 MstnPP cells were exposed to Thapsigargin (Tg; 400 nM) and Tunicamycin (Tm; 3 μg/ml) or solvent control DMSO. Cells were fixed 12 h post exposure and stained with **a** anti-APP antibody 6E10 (red) or **b** anti-Mstn-N antibody 6H12 (red). Images show co-staining with anti-Calreticulin antibody (green) and **c** perinuclear APP or **d** Mstn-N puncta or assemblies. Scale bars (A, B) 10 μm, (C, D and inset) 5 μm

To test if ER stress enhanced protein aggregation, we pharmacologically induced ER stress with standard concentrations of Thapsigargin (Tg) and Tunicamycin (Tm) to mimic the altered ER homeostasis implicated in sIBM [38, 61]. Tg induces ER stress by disrupting calcium homeostasis [62], while Tm induces the cellular UPR by inhibiting N-linked glycosylation in the ER [63]. No major cytotoxicity was observed for either drug in the given time and concentration (Supplementary Fig. 3a). Chemically induced ER stress did not overtly change intracellular APP staining independent of the antibody used for detection (Fig. 6a, c, Supplementary Fig. 3b). Tg-treated CCL 136 MstnPP cells appeared to exhibit more intense staining for Mstn. Aggregate-like structures immunoreactive with both amino- and carboxyl-terminal MstnPP antibodies were occasionally present (Fig. 6b, Supplementary Fig. 3c, insets). Interestingly, Tm treatment frequently induced large, aggregate-like assemblies that also stained positive with both antibodies (Fig. 6b, d, Supplementary Fig. 3c). Mstn assemblies also intensely stained for ER-resident chaperone Calreticulin (Fig. 6d). Some Tm-induced Mstn assemblies also associated with Lamp1, suggesting that aggregated MstnPP metabolites were subject to lysosomal degradation (Supplementary Fig. 4).

### Enhanced ER Stress Promotes Pro-Mstn and Propeptide Aggregation

**Fig. 7:**
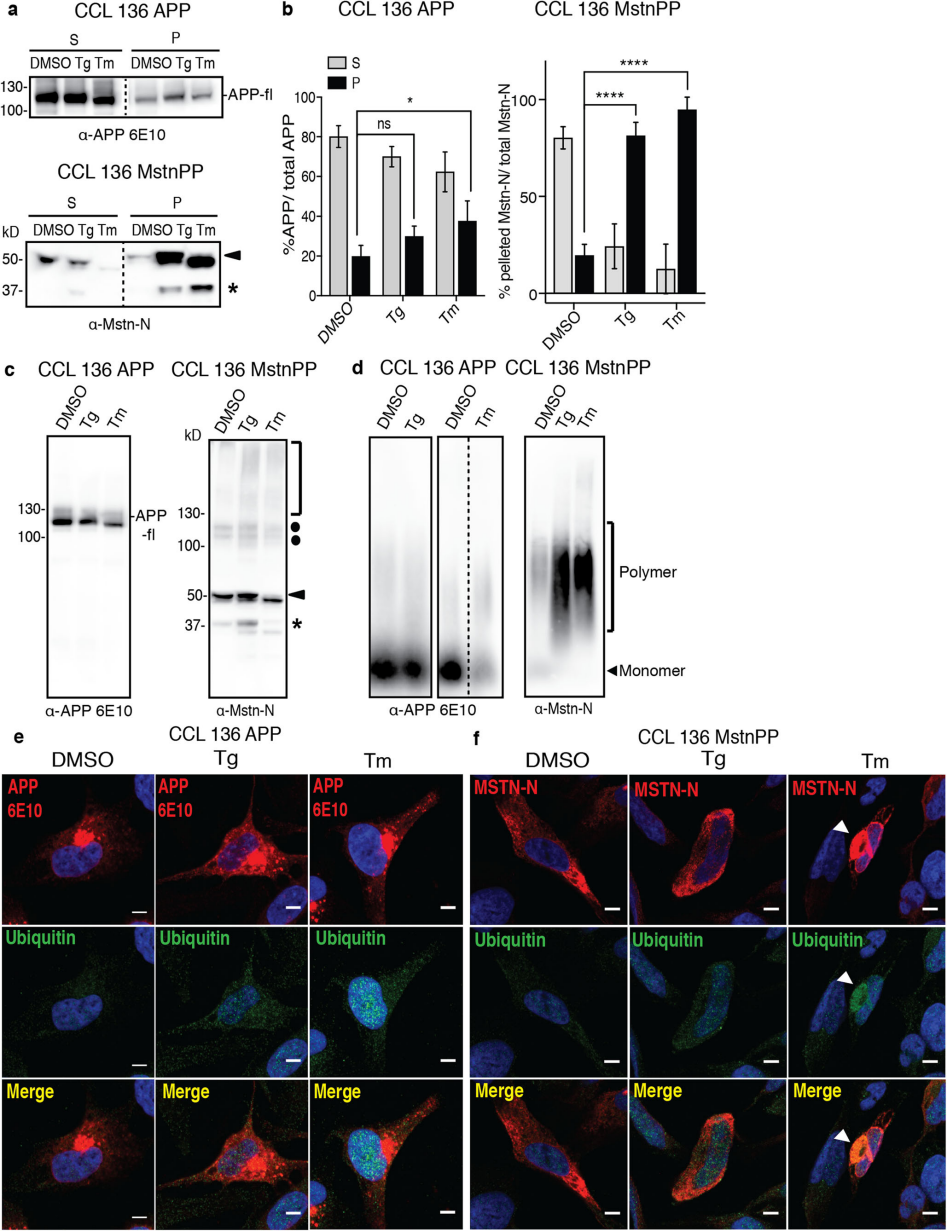
Ectopic ER stress increases Mstn-N aggregation. **a** Sedimentation assay of APP and Mstn-N in lysates of CCL 136 MstnPP and CCL 136 APP cells following ER stress induction. Lysates were subjected to ultracentrifugation at 150,000 g for 1 h and soluble (S) and insoluble fractions (P) were analyzed by western blot. Pro-Mstn (black arrowhead) and propeptide (star) are indicated. **b** Quantitative analysis of Mstn-N and APP in soluble and insoluble fractions of cells exposed to Tg or Tm (shown in **a**). Combined signals from soluble and insoluble fractions of the same samples were set as 100%. Bars represent mean values ± SD. Statistical analysis was performed using one-way ANOVA. Significant changes are indicated by asterisks. ****p < 0.0001, *p < 0.05, ns = not significant (*n* = 4). **c** Glutaraldehyde crosslinking reveals increased multimerization of Mstn-N post ER stress induction. Monomeric pro-Mstn (black arrowhead), monomeric propeptide (star), pro-Mstn dimers with intact disulfide bonds (dots), and high molecular weight species (parenthesis) are indicated. **d** SDD-AGE analysis demonstrates increased SDS-resistance of Mstn-N in cells upon chemical ER stress induction. **e**, **f** Co-localization of Mstn-N assemblies with Ubiquitin. Cells were exposed to ER stress (400 nM Tg or 3 μg/ml Tm) for 12 h and subsequently stained with **e** anti-APP (6E10), **f** anti-Mstn-N (6H12) (red) and anti-ubiquitin (green) antibodies. Arrows mark Mstn-N deposits co-staining for ubiquitin. Nuclei were stained with Hoechst. Scale bars 5 μm

To explore if enhanced ER stress correlated with protein insolubility, we biochemically assessed protein aggregation following treatment with Tg or Tm. Sedimentation assays revealed no significant effect of Tg treatment and only a slight increase in APP insolubility upon Tm treatment (Fig. 7a, b). Full-length APP was mainly present in the soluble fraction (Fig. 7a). A low amount of sedimented full-length APP in the insoluble fraction has been reported previously and has been attributed to an association with the cytoskeleton [64]. By contrast, elevated ER stress significantly increased the amount of insoluble pro-Mstn and propeptide following ER stress induction (Fig. 7a). More than 81% of pro-Mstn/propeptide became insoluble after ER stress induction compared to only 19.6 ± 5.7% insoluble protein in DMSO controls (Fig. 7b). Glutaraldehyde crosslinking of cell lysates was performed to test for the presence of higher-order protein assemblies upon chemical induction of ER stress. A slight increase in diffuse high molecular weight polymers was observed for Mstn-N under ER stress conditions (Fig. 7c, parenthesis). No high molecular weight assemblies were observed for APP. SDD-AGE analysis confirmed that experimentally induced ER stress increased the ratio of SDS-resistant polymeric to SDS-soluble monomeric Mstn-N species (Fig. 7d). 6E10-immunoreactive APP or cleavage products thereof remained predominately monomeric. Thus, elevated ER stress increases sequestration of pro-Mstn and propeptide into SDS-stable aggregates.

Misfolded ER-resident proteins are usually tagged via ubiquitin and sequestered for degradation by the ubiquitin proteasome system [23]. Ubiquitin and APP co-localization was hardly observed in cells overexpressing APP under any treatment (Fig. 7e). By contrast, large Tm-induced Mstn assemblies were found to be immunoreactive for ubiquitin (Fig. 7f). We conclude that enhanced ER stress can trigger the sequestration of pro-Mstn/propeptide into large ubiquitinated assemblies.

### Enhanced ER Stress Impairs Secretion of Pro-Mstn, Propeptide, and Mstn Growth Factor

**Fig. 8:**
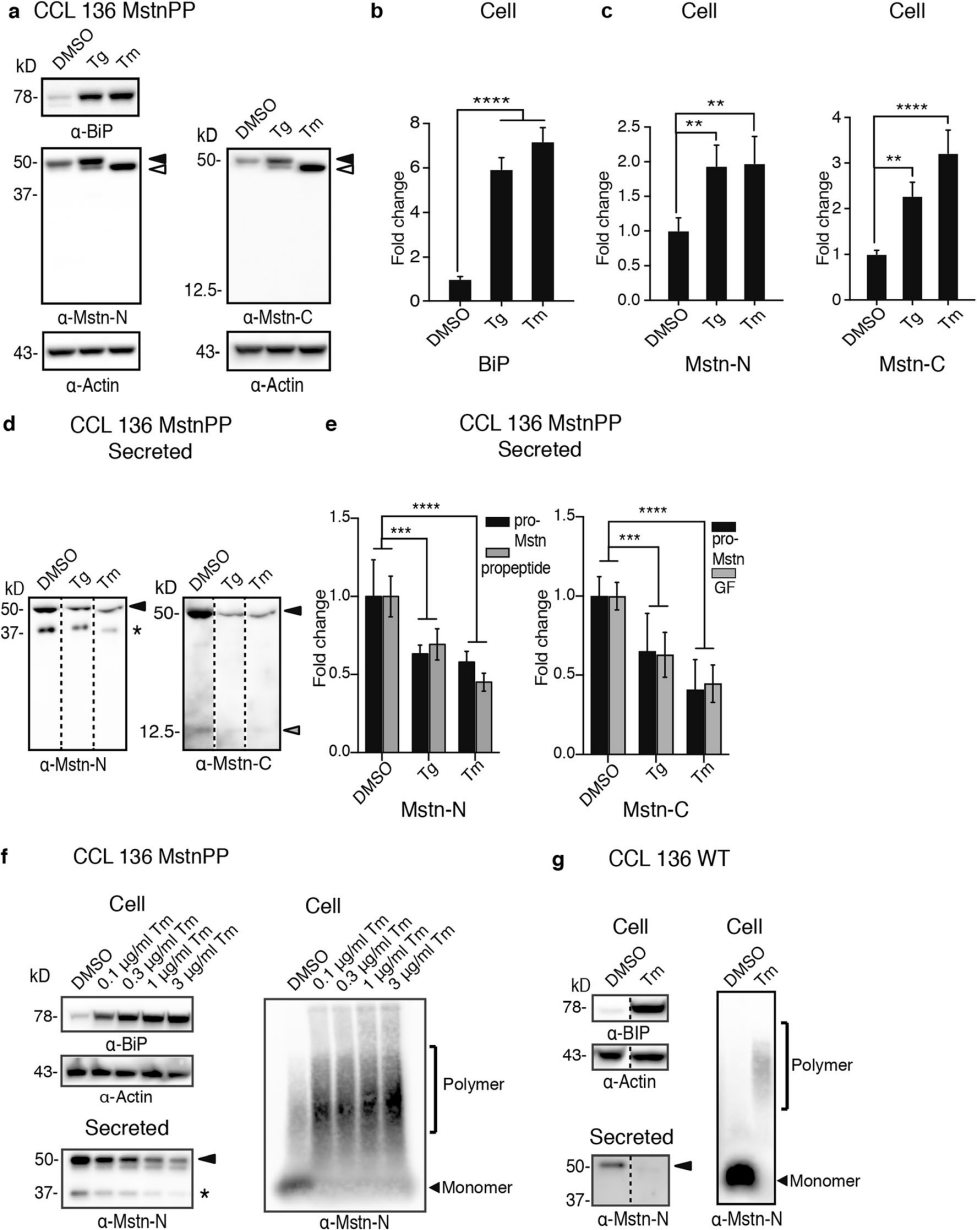
ER stress decreases secretion of pro-Mstn, propetide, and GF. **a** Mstn-N and -C levels following ER stress induction in CCL 136 MstnPP cells. The left blot was probed for Mstn-N, Actin, and BIP. The right blot (same samples as on left blot) was probed for Mstn-C and Actin. Pro-Mstn (black arrowhead) and unglycosylated Pro-Mstn due to Tm treatment (white arrowhead) are indicated. Note that MstnPP was expressed under the control of the CMV promoter and thus might not respond to ER stress-induced transcriptional changes as demonstrated before [65]. **b** Quantification of fold change in BIP (*n* = 4). **c** Quantification of Mstn-N and Mstn-C (*n* = 4). **d** Reduced secretion of MstnPP metabolites upon ER stress induction. Conditioned medium of ER stress induced cells was probed for Mstn-N (6H12) and Mstn-C (AF-788). Pro-Mstn (black arrowhead), GF (gray arrowhead) and propeptide (star) are indicated. **e** Fold change in secreted Mstn levels produced upon chemical ER stress (*n* = 6). Secreted samples were normalized to protein concentration in respective cell lysates. Statistical analysis was performed using one-way ANOVA. Significant changes are indicated by asterisks. ****p < 0.0001, ***p < 0.001, **p < 0.01. **f** Dose-dependent decrease in pro-Mstn and propeptide secretion upon Tm treatment. Cells were treated with different concentrations of Tm for 12 h to induced ER stress, as revealed by BIP increase. Medium was tested for the presence of pro-Mstn and propetide. Formation of SDS-resistant Mstn-N was confirmed using SDD-AGE. **g** ER stress reduces MstnPP metabolite secretion in wildtype CCL 136 cells. Wildtype cells were exposed to 3 μg/ml Tm for 12 h. Increased BIP levels confirm induction of ER stress. Culture medium was subsequently assessed for MstnPP metabolites (left panel). CCL 136 wildtype cells exposed to DMSO or 3 μg/ml Tm were assessed for the presence of SDS-resistant MstnPP metabolites using SDD-AGE

We next determined if chemically induced ER stress increased levels of APP and MstnPP and cleavage products thereof. Levels of full-length APP appeared only slightly altered, except for significant reduction by Tm detected by 6E10 (Supplementary Fig. 5a–c). Mstn-N and Mstn-C levels were significantly increased in CCL 136 MstnPP cell lysates upon Tg or Tm treatment (Fig. 8a–c), in agreement with the increase in anti-Mstn immunofluorescence (Fig. 6 and Supplementary Fig. 3c). Retention of proteins in the ER could potentially impair their subsequent secretion. Quantitative ELISA analysis revealed a slight but insignificant decrease in Aβ-42 secretion after treatment of CCL 136 APP cells with Tg (Supplementary Fig. 5d). A significant reduction in secreted Aβ-42 was observed following Tm treatment. Western blot analyses of secreted Mstn-N and Mstn-C demonstrated reduced Mstn species in conditioned medium of CCL 136 MstnPP cells following chemical ER stress induction (Fig. 8d). Quantitative analysis of western blot signals confirmed significantly reduced levels of secreted pro-Mstn, propeptide, and GF (Fig. 8e). Reduced secretion of MstnPP metabolites was also observed when cells were exposed to up to 30×-fold less Tm, a concentration that still induced BIP expression (Fig. 8f, left panel). The reduction of MstnPP metabolite secretion was dose dependent. Reduced secretion correlated with increased levels of SDS-resistant MstnPP metabolites in the cell lysate (Fig. 8f, right panel). An increase of SDS-resistant Mstn-N was also observed under mild chronic ER stress, when CCL 136 MstnPP cells were exposed to only 10 ng/ml for 7 d (Supplementary Fig. 6). Importantly, reduced secretion of MstnPP metabolites was also observed when ER stress was induced in wildtype CCL 136 cells (Fig. 8g). Less MstnPP metabolites were secreted upon ER stress compared to DMSO-treated control cells (Fig. 8g, left panel). Concomitantly, ER stress induction also resulted in SDS-resistant MstnPP metabolites in wildtype cells, as assessed by SDD-AGE (Fig. 8g, right panel). Thus, contrary to previously anticipated, ER stress results in MstnPP metabolite aggregation and reduces the secretion of Mstn GF.

## Discussion

Aberrant protein deposition plays a prominent role in sIBM. A diverse set of proteins has been found to accumulate within inclusions or around vacuoles in sIBM skeletal muscle [66]. While the aggregation state of many of these proteins has not been tested biochemically, congophilic inclusions and congophilic vacuole boundaries clearly argue for aberrant folding of at least some of them. The exact proteins that aberrantly fold into amyloid in sIBM are still a matter of debate [67]. The main reasons for this are the heterogeneity of proteins accumulating in inclusions and the inability of histological amyloid dyes to pin-point the amyloid-forming protein. Previous studies have demonstrated intramyofiber accumulation of proteins immunoreactive with antibodies against APP [12–14, 49] and MstnPP [34], among others [66]. Our analysis confirms the presence of anti-MstnPP immunoreactive inclusions and further reveals deposition of Mstn-N metabolites inside and lining the borders of vacuoles in sIBM muscle. Histological examinations with antibodies directed against the amyloidogenic Aβ region (such as 6E10) previously suggested that Aβ peptides deposit in sIBM skeletal muscle [21, 34, 38, 59]. However, as this region is also part of APP, immunohistochemical discrimination of APP and its metabolites is not possible [67]. Our finding that the inclusions in sIBM muscle biopsies also stain with antibodies against the APP aminoterminus is consistent with reported full-length APP deposition in sIBM [12, 68]. Intramyofiber deposition of full-length APP was also recently demonstrated in a mouse model of systemic amyloid disease overexpressing mutant gelsolin in the secretory pathway [69].

Intramuscular accumulation of diverse proteins in sIBM could also reflect aberrant protein retention in specific subcellular compartments. Of note, our cellular model of APP overexpression produces very little Aβ peptides or insoluble APP metabolites, yet cells contain APP positive vesicles clustering in the densely packed perinuclear vesicle cloud [55] which may be easily confused with Aβ protein aggregates. In depth biochemical analyses will help to determine the aggregation state of APP metabolites in sIBM or cellular models thereof. In conclusion, while we cannot exclude Aβ deposition in sIBM myofibers, the presence of APP immunoreactivity in immunohistochemical specimen could also reflect retention of full-length APP (rather than Aβ peptides) in specific subcellular compartments.

Further, we show that sIBM rimmed vacuoles stain strongly with antibodies against the ER resident chaperone Calreticulin, along with APP and MstnPP metabolites, supporting the idea that the ER is involved in vacuole formation in the disease [21, 48, 65, 70]. It is tempting to speculate that ER stress [26, 28, 34, 49] and intraluminal accumulation of secretory proteins trigger sIBM pathology, followed by dysregulation of proteasome and autophagy due to misfolded protein overload [30, 31, 71–73]. Both age-related increase in ER stress or pro-inflammatory cytokines could increase expression of aggregation-prone proteins in the secretory pathway [38]. Importantly, disruption of ER homeostasis due to misfolding of a specific protein might subsequently affect trafficking of other secretory proteins. For example, overexpression of secretory proteins APP or mutant gelsolin in murine skeletal muscle is sufficient to trigger multiprotein deposition and rimmed vacuole formation reminiscent of sIBM [69, 74].

In this study, we carefully characterized the processing and aggregation states of MstnPP and APP in human skeletal muscle cells and explored the influence of ER stress on the posttranslational processing and trafficking within the secretory pathway. In line with previous studies, MstnPP was posttranslationally processed into pro-Mstn, propeptide, and Mstn GF, with pro-Mstn being the most abundant MstnPP metabolite generated by muscle cells [47]. CCL 136 cells overexpressing APP secreted only very limited amounts of Aβ-42 into the medium, suggesting that amyloidogenic processing plays only a minor role in this cellular model.

Here, we demonstrate that an overload of MstnPP protein causes ER stress. Overexpression of MstnPP caused ER retention of Mstn propeptide and/or pro-Mstn and their spontaneous aggregation, suggesting that intracellular MstnPP metabolites negatively affect ER homeostasis. ER stress induction was likely due to misfolding of MstnPP metabolites, as overexpression of APP in the secretory pathway was ineffective at inducing ER stress. Pharmacologically aggravated ER stress enhanced sequestration of propeptide-containing MstnPP species into large intracellular aggregates that were often ubiquitinated, arguing that ER stress and MstnPP metabolite misfolding are interconnected. MstnPP metabolite aggregation following chemical ER stress induction was not due to artificial overexpression of the protein, as SDS-stable Mstn assemblies were also induced in wildtype cells expressing only very low levels of endogenous MstnPP. The finding that MstnPP metabolites assembled into SDS-resistant polymers is consistent with in vitro studies on amyloid formation of recombinant pro-Mstn under physiological buffer conditions [36]. By contrast, APP and its metabolites were abundant in post-ER compartments. Chemically induced ER stress neither altered the cellular location nor majorly influenced the aggregation state of APP metabolites. Thus, ER stress severely affected the aggregation and trafficking of MstnPP metabolites in our cellular model, while it left APP relatively unaffected.

Importantly, aggregation of MstnPP metabolites impaired secretion of pro-Mstn in both wildtype and MstnPP overexpressing cells. Thus, expression levels of MstnPP and secreted Mstn GF levels are not necessarily related. Stressful conditions and declining proteostasis could impair proper folding of MstnPP metabolites and thereby reduce the amount of secreted pro-Mstn and Mstn GF. Whether MstnPP plays a causal role in sIBM remains to be determined. The findings that ER stress and proteasomal dysfunction are features of sIBM [21, 22], pro-Mstn/propetide deposit with ER markers in sIBM muscle fibers and form highly stable aggregates upon ER stress ex vivo suggest that aberrant MstnPP processing contributes to the progression of sIBM. Interestingly, a recent study reported significantly reduced MsntPP mRNA levels in sIBM muscle and reduced serum Mstn GF concentrations in some but not all patients [33]. In another study, pro-Mstn protein levels were elevated in sIBM muscle [34]. As MstnPP metabolite deposition is evident in sIBM muscle biopsies, it is possible that downregulation of MstnPP expression is a consequence of aberrant MstnPP processing. Further studies are necessary to characterize MstnPP transcription and protein aggregation during disease progression.

Aberrant MstnPP processing could also play a role during aging. Interestingly, several studies suggest that muscle MstnPP mRNA levels increase with age [75–77]. In a cohort of old cattle showing signs of sarcopenia, age-related myopathic features reminiscent of sIBM, including Congo Red positive deposits and protein accumulations immunoreactive with anti-APP antibody 6E10 or anti-Mstn-C antibody, have been observed [78].

It is tempting to speculate that aberrant aggregation of MstnPP and its metabolites have downstream consequences on muscle metabolism also in sIBM. Recently, a late-phase clinical trial of the ActRIIB receptor antibody BYM338 [79] in sIBM patients failed to demonstrate efficacy, suggesting that ActRIIB receptor signaling is not a major contributor to muscle atrophy in sIBM (https://www.morphosys.de). The reported improvement of functional outcome in sIBM patients undergoing follistatin gene therapy implicates that beneficial effects of follistatin were elicited by alternative signaling pathways [80]. Our data strongly suggest that MstnPP expression does not necessarily correlate with increased levels of secreted Mstn GF or its precursors. Instead, ER homeostasis imbalance can cause intracellular MstnPP metabolite aggregation and thereby impair Mstn GF secretion. Targeting ER stress might therefor represent a promising route for therapeutic intervention in sIBM. Indeed, arimoclomol, a drug activating the cellular heat shock response and reducing ER stress ameliorated disease pathology and improved muscle function in an animal model with sIBM pathology [20].

Recent studies argue that serum level of Mstn GF decrease with age in humans and other mammals [81–85], even though MstnPP mRNA might be upregulated [75–77]. As proteolytic processing of MstnPP and complex posttranslational regulation control the liberation of biologically active Mstn GF, careful evaluation of Mstn processing and secretion is required, using immunoreagents that do not cross-react with other TGF-β GFs or immunoglobins [84]. Moreover, careful assessment of Mstn GF biological activity in serum and muscle is needed to fully understand the role of MstnPP upregulation in degenerative muscle diseases and aging.

## Electronic Supplementary Material

ESM 1 (PDF 9732 kb)
