# Supplementary material for: Endoplasmic Reticulum Stress Induces Myostatin High Molecular Weight Aggregates and Impairs Mature Myostatin Secretion
Source: Mol Neurobiol. 2018 Mar 15;55(11):8355–73. doi: 10.1007/s12035-018-0997-9 (PMC6153721; doi:10.1007/s12035-018-0997-9)

Supplementary Material

Endoplasmic reticulum stress induces Myostatin high molecular weight aggregates and impairs mature Myostatin secretion

Molecular Neurobiology

Rishibha Sachdev[a, b], Karin Kappes-Horn[c], Lydia Paulsen[a], Yvonne Duernberger[a], Catharina Pleschka[a], Philip Denner[a], Bishwajit Kundu[b], Jens Reimann[c] and Ina Vorberg[a,d *]

[b]Kusuma School of Biological Sciences, IIT Delhi, Hauz Khas, New Delhi 110016, India

[c]Department of Neurology, University of Bonn Medical Center, 53127 Bonn, Germany

[d]Department of Neurology, Rheinische Friedrich-Wilhelms University of Bonn, 53127 Bonn, Germany

[*]Corresponding author: Phone: 0049 228 43302560; Email: ina.vorberg@dzne.de; ORCID: 0000-0003-0583-4015

Supplementary Table. Patient data.

Age at biopsy (years)	Muscle	Histology	Gender
73	unknown	sIBM	female
66	vastus	sIBM	male
73	vastus	sIBM	male
59	vastus	sIBM	male
68	vastus	sIBM	female
67	vastus	sIBM	male
67	deltoid	sIBM	male
68	vastus	sIBM	male
79	vastus	sIBM	male
65	biceps brachii	sIBM	male
63	unknown	sIBM	male
50	tibialis anterior	neurogenic	male
14	vastus	neurogenic	male
37	biceps	neurogenic	male
59	vastus	normal	female
54	vastus	normal	female
59	vastus	normal	male
51	vastus	normal	male
55	vastus	normal	male
21	vastus	normal	male

Supplementary Figures

Fig. S1. N-linked glycosylation of APP and MstnPP metabolites. Lysates of CCL 136 APP and MstnPP cells were left untreated or were incubated with PNGase F to remove N-linked carbohydrates. Proteins were heated to 100 °C prior to deglycosylation. Mstn-N was detected using antibody 6H12. Actin served as a loading control. Pro-Mstn (black arrowhead) and propeptide (star) are indicated.

Fig. S2. Co-localization of APP and Mstn-N with ER markers BIP and Calnexin. Co-staining of **(A)** CCL136 APP with antibody 6E10 (red); **(B)** CCL136 MstnPP with antibody Mstn-N (6H12, red) and BiP, Calnexin and LC3 antibodies (green). Nuclei were stained with Hoechst. Scale bars: 5 µm.

Fig. S3. Ectopic ER stress does not affect cell viability. (A) Cell viability upon exposure to ER stress inducers. CCL 136 WT, APP or MstnPP were exposed to ER stressors for 12 h. Mitochondrial activity was subsequently assessed by XTT assay. Significant changes are indicated by asterisks. (n= 4; ANOVA with Dunnett's multiple comparison test; ****$p < 0.0001$, *$p < 0.05$ and ns= not significant). Cells were exposed to Tg or Tm for 12 hours and cellular distribution of **(B)** APP immunoreactive with aminoterminal antibody 22C11 and **(c)** MstnPP metabolites detected by anti-MstnPP antibody directed against carboxyterminal MstnPP (AF-788, α-Mstn-C) was assessed. Nuclei were stained with Hoechst. Scale bars: 10 µm, in insets: 5 µm.

Fig. S4. Co-localization of Tm-induced Myostatin assemblies with Lamp1. Cell populations were exposed to Tg or Tm for 12 h and subsequently stained with

antibodies against **(A, C)** APP (6E10) or **(B, D)** Mstn-N (6H12) (red) and markers for Golgi (Giantin) and lysosomes (Lamp1) (green). Nuclei were stained with Hoechst. Scale bar: 5 μm.

Fig. S5. (A) Enhanced ER stress does not increase APP protein levels or Aβ-42 secretion. BIP and APP levels following ER stress induction. CCL 136 APP cells were exposed to Tg, Tm or solvent control DMSO for 12 h and cell lysates tested by western blot using antibodies against APP (6E10), Actin and BIP. The same samples were probed for APP (22C11) and Actin on a separate western blot. **(B)** Quantification of fold change in BIP and APP levels following ER stress induction (n=4). **(C)** Detection of cell-associated and secreted Aβ-42 upon ER stress induction by ELISA. CCL 136 APP cells were exposed to the above chemicals for 12 h and Aβ-42 levels in cell lysates and conditioned medium were measured (n=3).

Fig. S6. Prolonged low-level ER stress induces aggregation of MstnPP metabolites. CCL 136 MstnPP cells were exposed to 10 ng/ml Tm or DMSO for 7 d. Cell lysates were assessed for BIP and MstnPP levels by western blot. Actin served as a loading control. Note that at this concentration, Tm only partially impaired pro-Mstn glycosylation (left panel). Low-level treatment of CCL 136 MstnPP cells resulted in formation of SDS-resistant Mstn-N assemblies as assessed by SDD-AGE (right panel).

Supplementary Figure 1

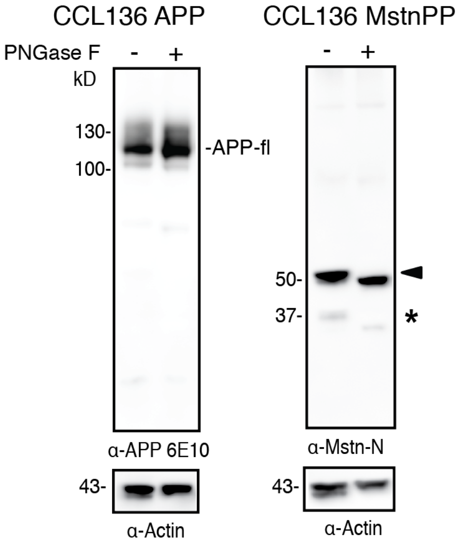

Supplementary Figure 2

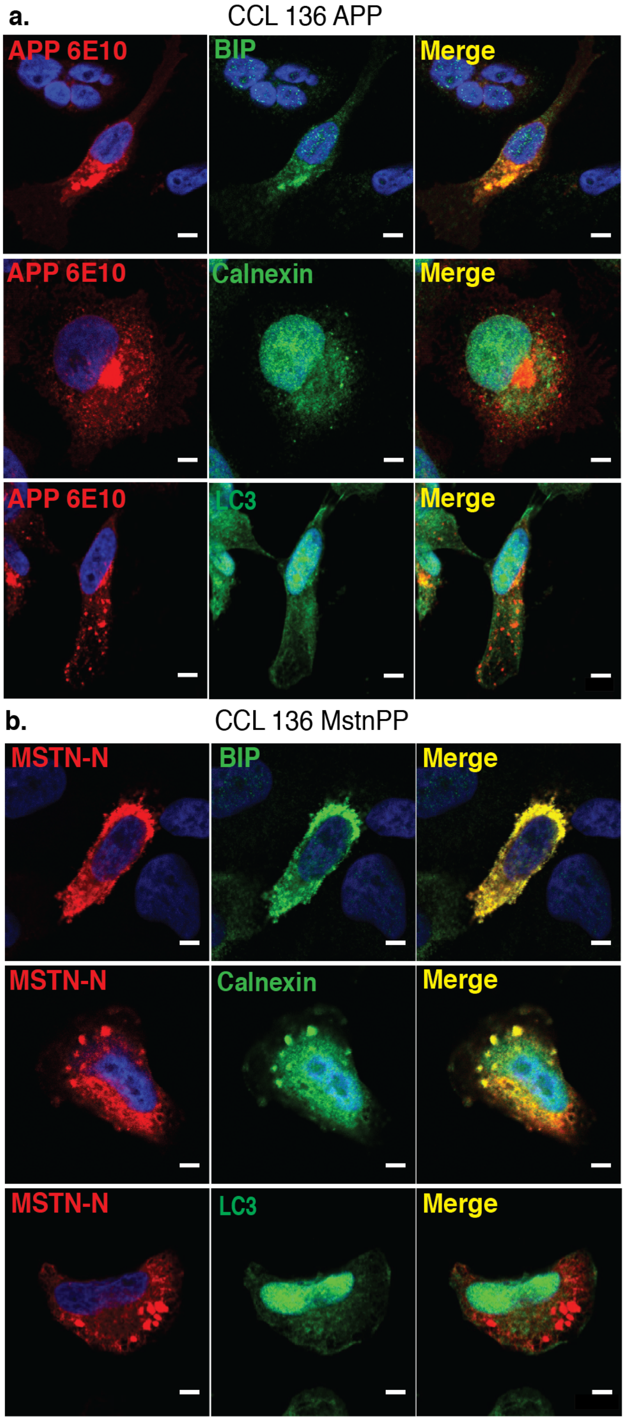

Supplementary Figure 3

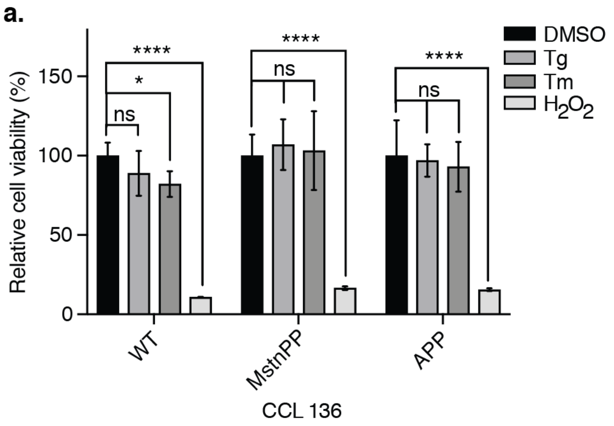

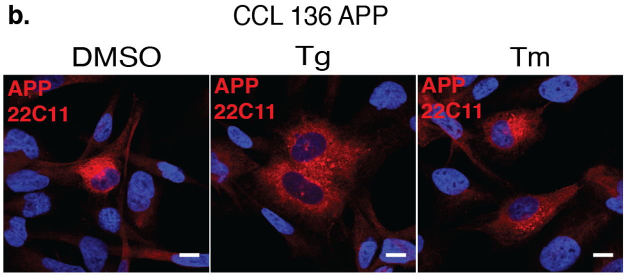

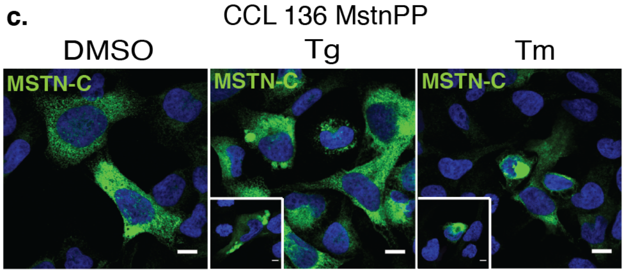

Supplementary Figure 4

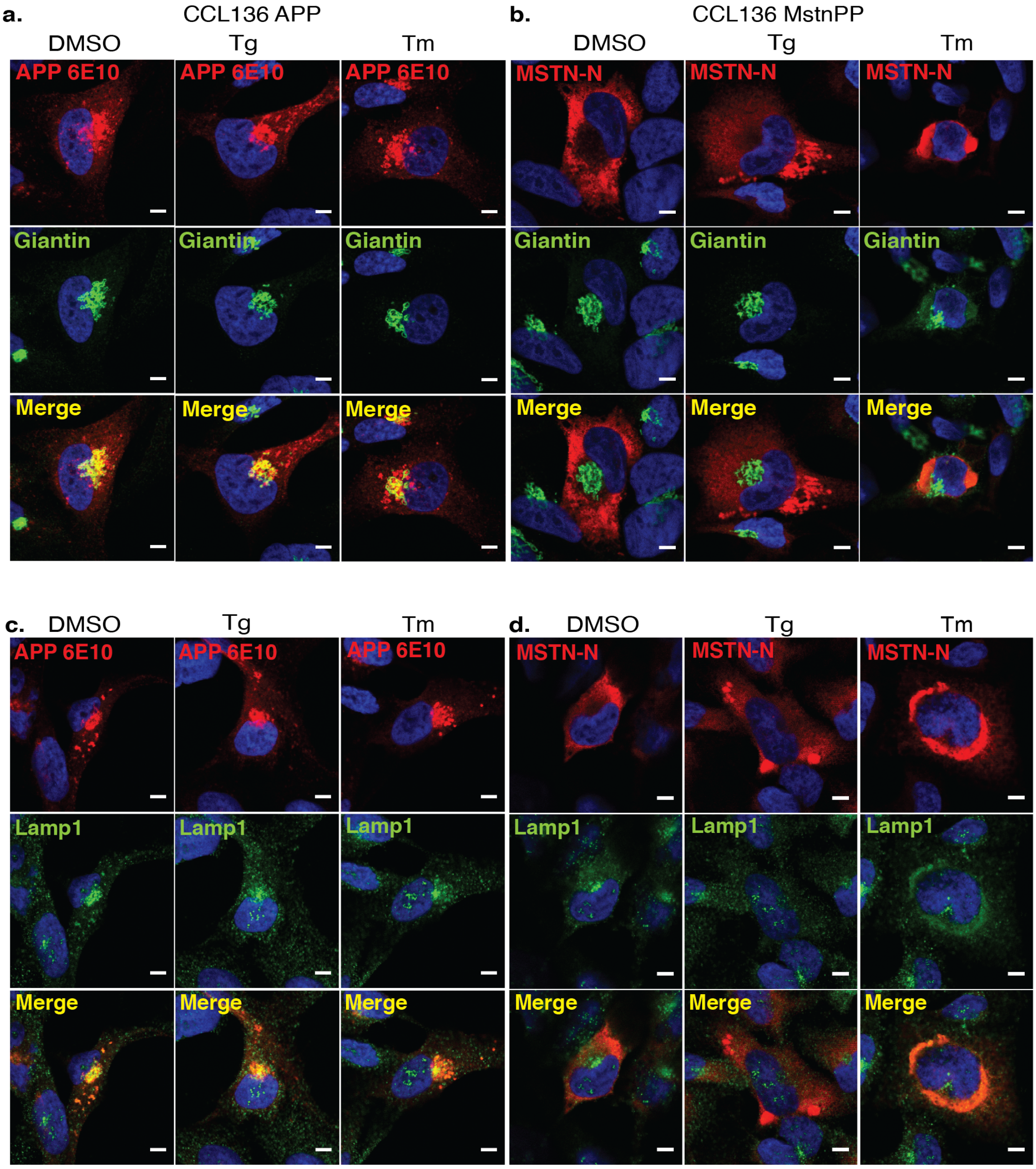

Supplementary Figure 5

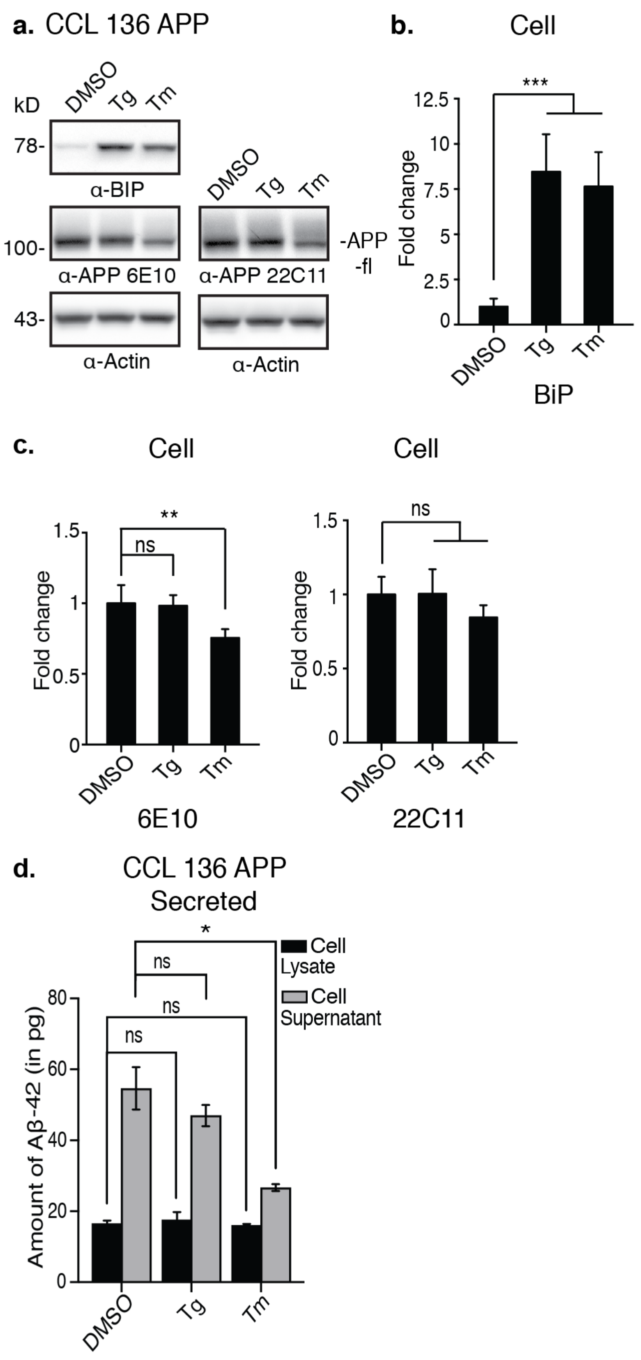

Supplementary Figure 6

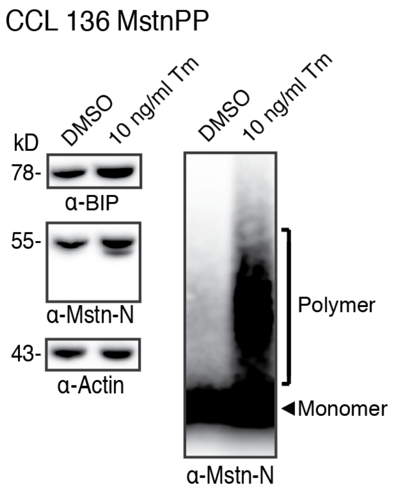

Supplement: Supplementary file 1 — (PDF 9732 kb) [file 12035_2018_997_MOESM1_ESM.pdf]
